# Inhibition of CHK1 sensitizes Ewing sarcoma cells to the ribonucleotide reductase inhibitor gemcitabine

**DOI:** 10.18632/oncotarget.18776

**Published:** 2017-06-28

**Authors:** Kelli L Goss, Stacia L Koppenhafer, Kathryn M Harmoney, William W Terry, David J Gordon

**Affiliations:** ^1^ Department of Pediatrics, Division of Pediatric Hematology/Oncology, University of Iowa, Iowa City, Iowa 52242, USA

**Keywords:** Ewing sarcoma, ribonucleotide reductase, gemcitabine, CHK1, replication stress

## Abstract

Ewing sarcoma is a bone and soft tissue sarcoma that occurs in children and young adults. The EWS-FLI1 gene fusion is the driver mutation in most Ewing sarcoma tumors and functions, in part, as an aberrant transcription factor. We recently identified that Ewing sarcoma cells are sensitive to inhibition of ribonucleotide reductase (RNR), which catalyzes the formation of deoxyribonucleotides from ribonucleotides. In this report, we show that Ewing sarcoma cells are sensitive to treatment with clofarabine, which is a nucleoside analogue and allosteric inhibitor of RNR. However, clofarabine is a reversible inhibitor of RNR and we found that the effect of clofarabine is limited when using a short (6-hour) drug treatment. Gemcitabine, on the other hand, is an irreversible inhibitor of the RRM1 subunit of RNR and this drug induces apoptosis in Ewing sarcoma cells when used in both 6-hour and longer drug treatments. Treatment of Ewing sarcoma cells with gemcitabine also results in activation of checkpoint kinase 1 (CHK1), which is a critical mediator of cell survival in the setting of impaired DNA replication. Notably, inhibition of CHK1 function in Ewing sarcoma cells using a small-molecule CHK1 inhibitor, or siRNA knockdown, in combination with gemcitabine results in increased toxicity both *in vitro* and *in vivo* in a mouse xenograft experiment. Overall, our results provide insight into Ewing sarcoma biology and identify a candidate therapeutic target, and drug combination, in Ewing sarcoma.

## INTRODUCTION

Ewing sarcoma is an aggressive bone and soft-tissue sarcoma that is defined by a recurrent chromosomal translocation between the *EWSR1* and *FLI1* genes [1]. Although Ewing sarcoma is currently treated with cytotoxic chemotherapy in combination with surgery and/or radiation, the EWS-FLI1 oncoprotein is an attractive therapeutic target because it is both required for tumorigenesis and specific for tumor cells [2–10]. But, in direct contrast to other oncogenes that can be directly inhibited using targeted therapies, EWS-FLI1 has proven to be a challenging target. Although work is currently underway to develop direct inhibitors of EWS-FLI1, an alternative therapeutic approach in Ewing sarcoma is to identify downstream targets of EWS-FLI1, or unique vulnerabilities incurred by the oncoprotein [11–19]. In previous work, we developed a human embryonic stem cell model of Ewing sarcoma and then used a gene expression signature based approach to identify ribonucleotide reductase (RNR) as a candidate therapeutic in Ewing sarcoma [20, 21].

RNR catalyzes the formation of deoxyribo-nucleotides from ribonucleotides and inhibiting RNR, by targeting either the RRM1 or RRM2 subunit of the heterodimeric enzyme complex, impairs DNA replication and causes replication stress [22, 23]. Notably, EWS-FLI1 has been implicated as a regulator of multiple aspects of the cellular response to genotoxic stress, although the mechanistic details remain to be elucidated [24]. For example, Ewing sarcoma cells are vulnerable to drugs that cause DNA damage during S-phase, including camptothecin analogs, PARP inhibitors, and cisplatin [25–31]. Furthermore, recent work from Nieto-Soler et al. showed, using DNA fiber analysis, that Ewing sarcoma cells exhibit elevated levels of endogenous DNA replication stress and are sensitive *in vitro* and *in vivo* to inhibitors of Ataxia Telangiectasia and Rad3-Related Protein (ATR), a kinase activated by DNA damage and impaired DNA replication [25].

Inhibition of RNR is known to cause cell cycle arrest and senescence in multiple types of cancer [32–34]. However, in Ewing sarcoma cells, in direct contrast to the other cell types we tested, inhibition of RNR causes cell cycle arrest and subsequent cell death with up-regulation of markers of apoptosis [21]. Notably, multiple inhibitors of RNR are currently used in clinical oncology [22, 23, 35]. For example, RRM1 can be targeted using both allosteric inhibitors (fludarabine and clofarabine) and catalytic inhibitors (gemcitabine) [22]. Similarly, iron chelators, (ciclopirox, triapine and deferoxamine) and free radical scavengers (hydroxyurea) inhibit RRM2 [22]. The dimerization of RRM1 and RRM2 can also be blocked using the small-molecule drug COH29, which is currently being tested in clinical trials [36, 37]. Although small-molecule inhibitors represent the primary strategy for RNR inhibition, siRNA-based approaches to target RNR are also being tested in clinical trials [38, 39].

In this report, we show that clofarabine, which is a nucleoside analogue and reversible inhibitor of RNR, induces apoptosis in Ewing sarcoma cells [40, 41]. However, the induction of apoptosis by clofarabine in Ewing sarcoma cells is ineffective when using short (6- hour) drug treatments because cells are able to recover and re-initiate DNA synthesis. In direct contrast, a single, 6-hour treatment with gemcitabine, an irreversible inhibitor of RNR, causes DNA replication stress, apoptosis, and cell death in Ewing sarcoma cells [42]. Moreover, we also found that inhibition of checkpoint kinase 1 (CHK1), the major regulator of the response to impaired DNA replication, significantly increases the toxicity of gemcitabine in Ewing sarcoma cells both *in vitro* and *in vivo* [43–45]. Overall, our results provide novel insight into Ewing sarcoma biology and identify a candidate therapeutic target in Ewing sarcoma.

## RESULTS

### Aphidicolin and clofarabine impair DNA replication and induce apoptosis in Ewing sarcoma cells

**Figure 1:**
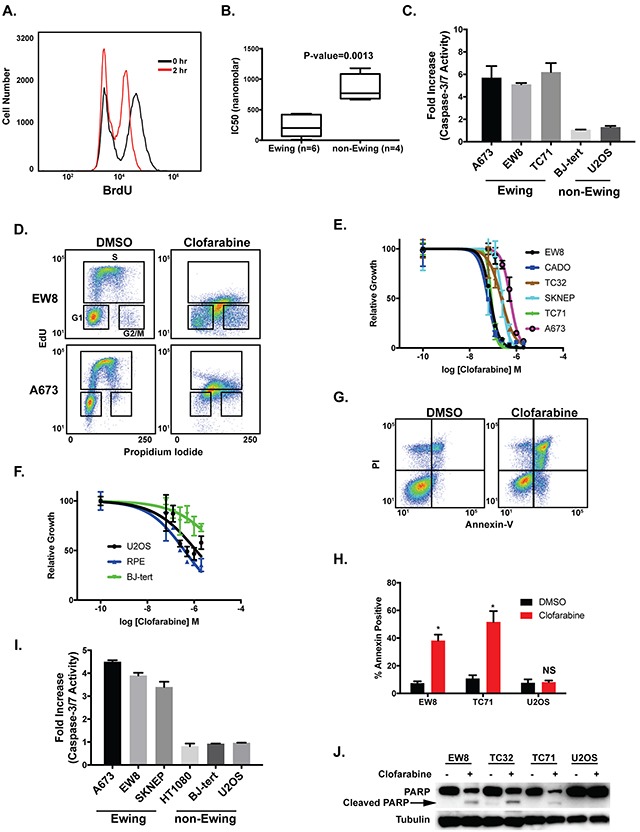
Aphidicolin and clofarabine impair the growth of Ewing sarcoma cells **(A)** Treatment of EW8 cells with aphidicolin (1 μM) for two hours decreases BrdU incorporation. **(B)** Box plots show the IC50 values for Ewing sarcoma (n=6) and control cell lines (n=4) treated with aphidicolin for 48 hours. Values between the 25th and 75th percentile are enclosed within the boxes and the whiskers encompass the smallest to largest values. **(C)** Fold increase in caspase-3/7 activation in Ewing sarcoma and control cells lines treated with aphidicolin (1 μM) for three days. Fold change is relative to cells treated with DMSO. Figures are representative of three independent experiments. Data represent mean ± SD of three technical replicates. **(D)** Cell cycle analysis with EdU and propidium iodide shows that treatment of Ewing sarcoma cell lines (A673 and EW8) with clofarabine (500 nM) results in a mixture of replicating and non-replicating S-phase cells. **(E)** Dose-response curves for six Ewing sarcoma cell lines treated with different concentrations of clofarabine for three days. Cell viability was assessed using the AlamarBlue Fluorescence Assay. **(F)** Dose-response curves for non-Ewing sarcoma cell lines treated with different concentrations of clofarabine for three days. For the dose-response experiments, the results are representative of two independent experiments. Error bars represent mean ± SD of three technical replicates. **(G)** Representative flow cytometry plot for Annexin-V and PI staining of EW8 cells treated with DMSO or clofarabine (100 nM) for two days. **(H)** Percentage of Annexin-V positive cells for two Ewing sarcoma cell lines and an osteosarcoma cell line (U2OS) treated with clofarabine (100 nM) for two days. Results are representative of two independent experiments. Error bars represent mean ± SD of two technical replicates. ^*^ P-value < 0.05. **(I)** Fold increase in caspase-3/7 activation in Ewing sarcoma and control cells lines treated with clofarabine (100 nM) for two days. Fold change is relative to cells treated with DMSO. Figures are representative of three independent experiments. Data represent mean ± SD of three technical replicates. **(J)** Western blot showing that treatment of Ewing sarcoma cells, but not U2OS osteosarcoma cells, with clofarabine (100 nM) results in cleavage of PARP-1.

In previous work, we identified that Ewing sarcoma cells are sensitive to iron chelators and other drugs that inhibit RNR [21]. Inhibition of RNR is known to deplete nucleosides and cause DNA replication stress [32–34]. To test whether Ewing sarcoma cells are sensitive to DNA replication stress caused by mechanisms other than inhibition of RNR, we treated Ewing sarcoma and control cell lines with aphidicolin, which is an inhibitor of DNA polymerase α and δ and a drug that is frequently used to synchronize cells in S-phase [46, 47]. Aphidicolin, as anticipated for an inhibitor of DNA polymerase, impaired DNA replication in Ewing sarcoma cells, as assessed using a BrdU-incorporation assay (Figure 1A). Treatment of Ewing sarcoma cell lines (n=6) with aphidicolin for 48 hours caused a significant reduction in growth (Figure 1B), with IC50 values ranging from 100 nM to 430 nM. In contrast, control cell lines (n=4), including HT1080 (fibrosarcoma), U2OS (osteosarcoma), BJ-tert (telomerase-immortalized fibroblasts) and RPE-tert (telomerase-immortalized epithelial) cells, were less sensitive to aphidicolin (Figure 1B). Furthermore, as shown in Figure 1C and Supplementary Figure 1, aphidicolin also caused cleavage of PARP-1 and activation of caspase-3/7, which are markers of apoptosis, in the Ewing sarcoma cells, but not the control cells.

Clofarabine is a second-generation nucleoside analogue and a potent inhibitor of both RNR and DNA polymerase α [40, 41]. Based on the sensitivity of Ewing sarcoma cells to inhibition of RNR and DNA polymerase we next tested whether clofarabine causes toxicity and apoptosis in Ewing sarcoma cells. Figure 1D and Supplementary Figure 2 demonstrate that treatment of Ewing sarcoma cells with clofarabine causes cell cycle arrest in S-phase, as assessed using EdU and propidium iodide cell cycle analysis. In a cell growth assay, Ewing sarcoma cells were more sensitive than the control cell lines, including U2OS, BJ-tert, and RPE-tert, to treatment with clofarabine (Figure 1E and 1F). Clofarabine also induced apoptosis in Ewing sarcoma cells, as assessed using annexin-V staining (Figure 1G and 1H), activation of caspsase-3/7 (Figure 1I), and cleavage of PARP-1 (Figure 1J). We also detected phosphorylation of checkpoint kinase 1 (CHK1), the major regulator of the response to impaired DNA replication, after treatment with clofarabine (Supplementary Figure 3) [43, 48, 49].

### Ewing sarcoma cells restart DNA replication after short (6-hour) treatments with clofarabine

**Figure 2:**
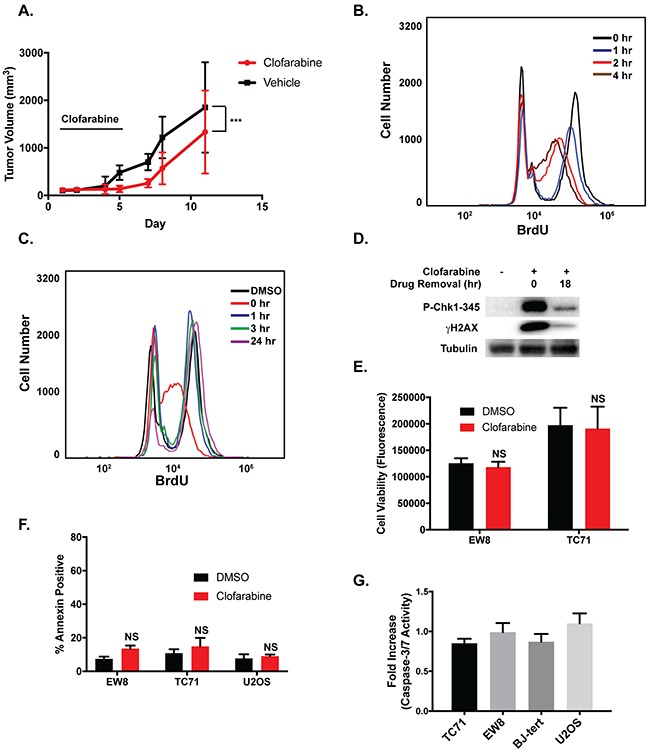
The effects of clofarabine on Ewing sarcoma cell growth are reversible **(A)** TC71 cells were engrafted in nude mice. After developing tumors, the mice were divided into two cohorts and treated with either vehicle or clofarabine (50 mg/kg) by oral gavage for five days (n=9 mice per group). The black bar indicates the days of drug administration. Tumor size was quantified using caliper measurements and tumor volumes were calculated using the equation volume = 0.5 × length × width^2^ (mean ± SD). All animals were sacrificed when a tumor reached 20 mm in any dimension. P-value was determined by 2-way ANOVA comparing the treatment curve to the vehicle curve. ^***^ P-value < 0.001. **(B)** Flow cytometry quantification of BrdU incorporation into the DNA of EW8 cells at different time points after the addition of clofarabine (500 nM). **(C)** EW8 cells were treated with clofarabine (500 nM) for 6 hours. BrdU incorporation was then quantified using flow cytometry at different time points after the removal of the drug. **(D)** EW8 cells were treated with clofarabine for 6 hours. Cell lysates were then collected and blotted for P-CHK1 and γH2AX at 0 hours and 18 hours after drug removal. **(E)** Ewing sarcoma cells were treated with clofarabine (500 nM) for six hours, followed by drug removal and additional incubation for 42 hours. Cell viability was then quantified using the AlamarBlue Fluorescence Assay. NS, not significant. **(F)** Percentage of Annexin-V positive cells for two Ewing sarcoma cell lines and an osteosarcoma cell line (U2OS) treated with clofarabine (500 nM) as described in **(E)**. Results are representative of two independent experiments. Error bars represent mean ± SD of two technical replicates. **(G)** Fold increase in caspase-3/7 activation in Ewing sarcoma and control cells lines treated with clofarabine (500 nM) for six hours followed by a 42-hour recovery period. Fold change is relative to cells treated with DMSO. Data represent mean ± SD of three technical replicates.

Based on the *in vitro* growth inhibition and apoptosis data, we next tested whether clofarabine could inhibit the growth of tumor cells in mouse xenograft experiments. NCr mice were subcutaneously injected with Ewing sarcoma (TC71) cells and allowed to develop measurable tumors. The mice were then treated with oral clofarabine (50 mg/kg) or vehicle daily for five days. Although the clofarabine treatment significantly decreased tumor size compared to vehicle this effect on tumor growth was modest and not sustained, which suggests that clofarabine has a cytostatic effect against Ewing sarcoma cells *in vivo* (Figure 2A). Consequently, while the *in vitro* clofarabine data support the critical role of RNR in Ewing sarcoma tumorigenesis, the *in vivo* xenograft experiment suggests that the clinical utility of this drug, when administered in a 5-day dosing regimen, may be limited.

Clofarabine is a reversible inhibitor of RNR and has a half-life of ~5-7 hours *in vivo* so we next asked whether more prolonged, sustained inhibition of RNR, similar to that achieved in the *in vitro* assays (Figure 1), is required to induce apoptosis and toxicity in Ewing sarcoma cells [41, 50, 51]. We used a BrdU incorporation assay to evaluate the reversibility of the effects of clofarabine on DNA replication in Ewing sarcoma cells. Although treatment of Ewing sarcoma cells with clofarabine resulted in a time-dependent decrease in BrdU incorporation (Figure 2B), the cells were able to resume DNA synthesis within one hour after clofarabine was removed from the assay (Figure 2C). The ability to re-initiate DNA synthesis after treatment with clofarabine correlated with reversal of phosphorylation of CHK1 and H2AX, markers of DNA replication stress and DNA damage (Figure 2D) [43, 52–54]. Removal of clofarabine after a 6-hour drug treatment also rescued Ewing sarcoma cells from the effects of the drug on cell viability (Figure 2E) and apoptosis, as assessed using annexin-V staining (Figure 2F) and caspase-3/7 activation (Figure 2G). In summary, these data suggest that induction of apoptosis in Ewing sarcoma cells by RNR inhibitors require an extended treatment duration and that the reversibility of clofarabine, in combination with its *in vivo* half-life of ~5-7 hours, may limit the effectiveness of this drug in a clinical setting.

### Gemcitabine is an irreversible inhibitor of RNR and causes apoptosis in Ewing sarcoma cells with short (6-hour) drug treatments

**Figure 3:**
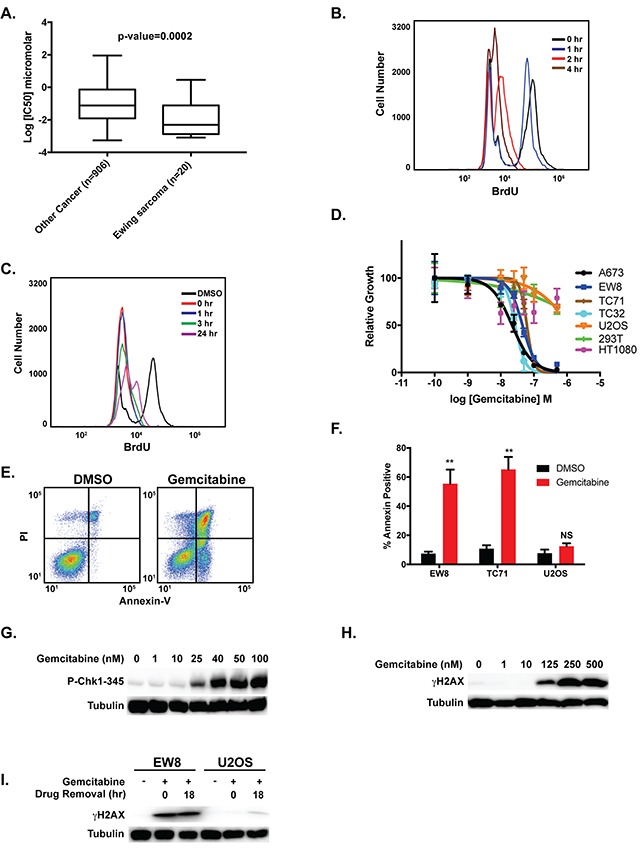
Gemcitabine impairs the growth of Ewing sarcoma cells **(A)** Analysis of Genomics of Drug Sensitivity in Cancer data shows that Ewing sarcoma cell lines are more sensitive to gemcitabine than other cancer cell lines. A Mann-Whitney test was performed to compare the drug sensitivity of Ewing sarcoma cell lines versus other cell lines. **(B)** Quantification of BrdU incorporation into the DNA of EW8 cells at different time points after the addition of gemcitabine (100 nM). **(C)** EW8 cells were treated with gemcitabine (100 nM) for 6 hours. BrdU incorporation was then quantified using flow cytometry at different time points after the removal of the drug. **(D)** Dose-response curves for four Ewing sarcoma cell lines and three non-Ewing sarcoma cell lines (U2OS, HEK-293T, and HT1080) treated with different concentrations of gemcitabine for 6 hours. Cell viability was then assessed 42 hours after drug removal using the AlamarBlue Fluorescence Assay. The results are representative of two independent experiments. Error bars represent mean ± SD of three technical replicates. **(E)** Representative flow cytometry plot for Annexin-V and PI staining of EW8 cells treated with DMSO or gemcitabine (100 nM) for 6 hours followed by a 42-hour recovery period. **(F)** Percentage of Annexin-V positive cells for two Ewing sarcoma cell lines and an osteosarcoma cell line (U2OS) treated with gemcitabine as described in **(E)**. Results are representative of two independent experiments. Error bars represent mean ± SD of two technical replicates. **(G)** Western blot showing that treatment of EW8 cells with gemcitabine for 6 hours results in the dose-dependent phosphorylation of CHK1-345. **(H)** Western blot showing that treatment of EW8 cells with gemcitabine for 6 hours results in the dose-dependent phosphorylation of H2AX. **(I)** EW8 and U2OS cells were treated with 100 nM gemcitabine for 6 hours. Cell lysates were then collected and blotted for γH2AX at 0 hours and 18 hours after drug removal.

Gemcitabine is an irreversible inhibitor of the RRM1 subunit of RNR [42, 55, 56]. In previous work, we showed that Ewing sarcoma cells are more sensitive (IC50 range 2.4-10 nM) to gemcitabine than control cell lines using a standard dose-response assay with a 72-hour drug incubation [21]. Additionally, analysis of the Genomics of Drug Sensitivity in Cancer Project data (http://www.cancerrxgene.org/), which includes 20 Ewing sarcoma cell lines and >900 other cancer cell lines, demonstrated that Ewing sarcoma cells are significantly more sensitive to gemcitabine than other types of cancer (P-value=0.002) (Figure 3A) [57]. Similar to clofarabine, we used a BrdU assay to evaluate the reversibility of the effects of gemcitabine on DNA replication in Ewing sarcoma cells. Gemcitabine caused a time-dependent decrease in BrdU incorporation with near complete inhibition occurring after ~2-4 hours of drug treatment (Figure 3B). Removal of the gemcitabine after a 4-hour drug treatment resulted in sustained arrest of DNA replication (Figure 3B and 3C), in direct contrast to the results with clofarabine (Figure 2B and 2C). Similar results were obtained with U2OS osteosarcoma cells (Supplementary Figure 4). Furthermore, treatment of Ewing sarcoma cell lines with gemcitabine for 6 hours followed by drug removal and culture for an additional 42 hours resulted in a significant reduction in Ewing sarcoma cell growth (Figure 3D), with an IC50 of ~50 nM. This 6-hour gemcitabine treatment was also sufficient to induce apoptosis in the Ewing sarcoma cells, as assessed using annexin-V staining (Figure 3E and 3F) and caspase-3/7 activation (Supplementary Figure 5).

DNA replication stress results in the phosphorylation and activation of CHK1, which is a critical regulator of cell survival and the response to impaired DNA replication [45, 58–61]. When activated via phosphorylation by ATR, CHK1 promotes stabilization of stalled replication forks, suppresses the firing of replication origins, and prevents cells with damaged or incompletely replicated DNA from entering mitosis. Nieto-Soler et al. recently demonstrated that Ewing sarcoma cell lines are characterized by decreased DNA replication fork progression, indicative of replication stress, and elevated levels of CHK1 [25]. We detected dose-dependent phosphorylation of CHK1 in Ewing sarcoma cells treated with gemcitabine (Figure 3G) for 6 hours. Treatment with gemcitabine also caused of phosphorylation of H2AX, although this occurred at higher concentrations of gemcitabine than the phosphorylation of CHK1 (Figure 3H). Notably, in direct contrast to the results with clofarabine, the phosphorylation of γH2AX persisted after the removal of the gemcitabine in Ewing sarcoma cells (Figure 3I).

### Inhibition of CHK1 is synergistic with gemcitabine

**Figure 4:**
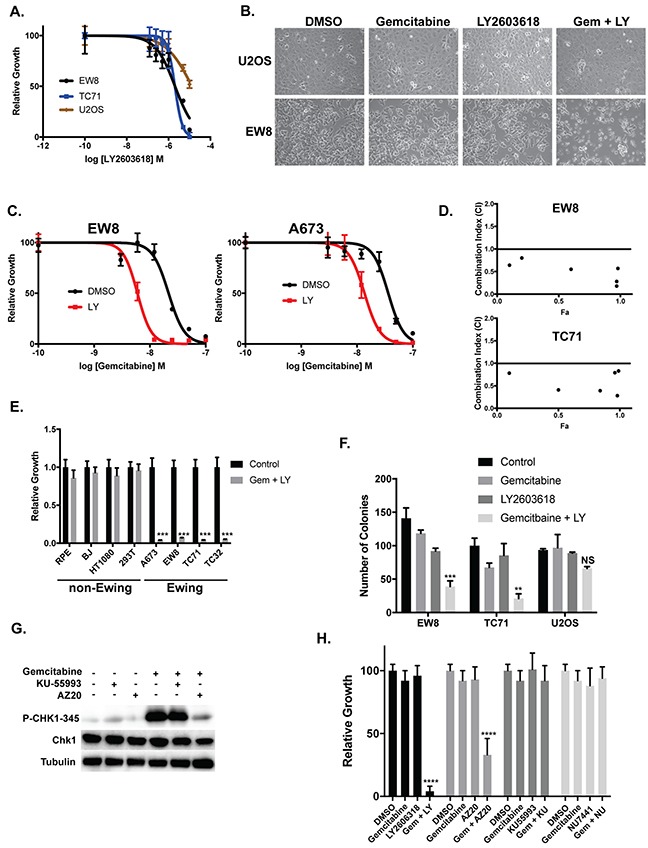
Ewing sarcoma cells are sensitive to inhibition of CHK1 **(A)** Dose-response curves for two Ewing sarcoma cell lines and an osteosarcoma cell line (U2OS) treated with different concentrations of a CHK1 inhibitor (LY2603618) for 6 hours. Cell viability was then assessed 42 hours after drug removal using the AlamarBlue Fluorescence Assay. The results are representative of two independent experiments. Error bars represent mean ± SD of three technical replicates. **(B)** Treatment of Ewing sarcoma cells with gemcitabine (10 nM) in combination with LY2603618 (250 nM) causes morphologic changes in Ewing sarcoma cells suggestive of cell death and apoptosis. **(C)** Dose-response curves for two Ewing sarcoma cell lines treated with different concentrations of gemcitabine in the presence or absence of LY2603618 (250 nM) for 6 hours. Cell viability was then assessed 42 hours after drug removal using the AlamarBlue Fluorescence Assay. The results are representative of two independent experiments. Error bars represent mean ± SD of three technical replicates. **(D)** EW8 and TC71 cells were treated with different concentrations of gemcitabine and LY2603618, using a constant drug ratio, for six hours after which the drugs were removed and cell viability was measured 42 hours later. Data were analyzed using the CompuSyn software. Combination Index (CI) versus Fraction Affected (Fa) plot shows the effect of the combination of gemcitabine and LY2603618. CI<0.9 indicates synergism. **(E)** Ewing sarcoma and non-Ewing sarcoma cell lines were treated with gemcitabine (10 nM) in combination with LY2603618 (250 nM) for 6 hours. Cell viability was then assessed 42 hours after drug removal using the AlamarBlue Fluorescence Assay. Results are representative of two independent experiments. Error bars represent mean ± SD of three technical replicates. ^***^ P-value < 0.001 **(F)** Treatment of Ewing sarcoma cells (EW8 and TC71), but not osteosarcoma cells, with the combination of gemcitabine (10 nM) and LY2603618 (250 nM) for 6 hours inhibits cell growth in colony formation assays. Results are representative of three independent experiments. Error bars represent mean ± SD of three technical replicates. ^***^ P-value < 0.001, ^**^ P-value < 0.01 (1-way ANOVA, Dunnett's *post hoc* test). **(G)** Treatment of EW8 cells with gemcitabine in combination with an ATR inhibitor (AZ20), but not an ATM inhibitor (KU-55933), blocks the phosphorylation of CHK1-345. **(H)** EW8 cells were treated with gemcitabine (10 nM) in combination with drugs that target components of the DNA damage response pathway, including inhibitors of ATM (KU-55993; 500 nM), ATR (AZ20; 500 nM), DNA-PK (NU7441; 500 nM), and CHK1 (LY2603618; 500 nM). Cell viability was then assessed 42 hours after drug removal using the AlamarBlue Fluorescence Assay. Error bars represent mean ± SD of three technical replicates. ^****^ P-value< 0.0001 (1-way ANOVA, Dunnett's *post hoc* test).

Inhibition of CHK1 function, using either small molecule inhibitors or siRNA, is known to be synergistic with gemcitabine and other drugs that cause DNA replication stress [62–67]. Based on the increased level of replication stress in Ewing sarcoma cells and the known sensitivity of Ewing sarcoma cells to ATR inhibitors, we tested a CHK1 inhibitor (LY2603618) as a single agent to evaluate the effect of inhibition of CHK1 on the viability of Ewing sarcoma cells [25]. In a dose-response assay, Ewing sarcoma were sensitive to treatment with LY2603618, a potent inhibitor of CHK1, for 6 hours (Figure 4A) and 72 hours (Supplementary Figure 6) with IC50 values of ~2 μM and ~500 nM, respectively [62, 68–70]. To determine the concentration of LY2603618 required to inhibit CHK1 function we treated Ewing sarcoma cells with gemcitabine in combination with different concentrations of LY2603618 and then assessed CHK1 activation using immunoblotting for CHK1-Ser296, which is an auto-phosphorylation site in the CHK1 protein [71]. Supplementary Figure 7 shows that LY2603618 caused dose-dependent inhibition of CHK1-296 auto-phosphorylation, with maximum effect at drug concentrations > 200 nM.

We then treated Ewing sarcoma and osteosarcoma cells for 6 hours with 250 nM LY2603618 in combination with 10 nM gemcitabine, which is a gemcitabine concentration that does not affect cell viability when used in a 6-hour drug treatment (Figure 3D). The combination of the two drugs, but neither drug as a single agent, caused significant morphologic changes suggestive of cell death in the Ewing sarcoma cells, but not osteosarcoma cells (Figure 4B). Next, we treated two Ewing sarcoma cells lines for 6 hours with a range of doses of gemcitabine in the presence or absence of 250 nM LY2603618. Figure 4C shows that the addition of LY2603618 increased the sensitivity of two Ewing sarcoma cell lines to gemcitabine, with a ~5-fold reduction in IC50 values. We also used the method of Chou and Talalay to calculate a combination index (CI) to test if the combination of gemcitabine and LY2603618 was synergistic [72]. The combination of gemcitabine and LY2603618 demonstrated synergism (CI<0.9) in two Ewing sarcoma cell lines (EW8 and TC71) with CI values ranging from 0.18 to 0.83 (Figure 4D) [72]. We also treated additional Ewing sarcoma and control, non-Ewing sarcoma cell lines (RPE-tert, BJ-tert, HT1080, and HEK-293T) with 10 nM gemcitabine and 250 nM LY2603618 for six hours. In contrast to the Ewing sarcoma cell lines, none of the control cell lines demonstrated toxicity with the combination of gemcitabine and LY2603618 when the drugs were used in a single, 6-hour treatment (Figure 4E). Similarly, long-term clonogenic assays showed that a 6-hour treatment with 10 nM gemcitabine and 250 nM LY2603618 significantly inhibited the growth of Ewing sarcoma (EW8 and TC71), but not osteosarcoma, cells (Figure 4F).

Ataxia Telangiectasia and Rad3-related protein (ATR) is the canonical upstream kinase and activator of CHK1, although alternative regulators of CHK1 include ATM and DNA-PK (Figure 4H) [43, 59, 61, 73, 74]. Figure 4G shows that the phosphorylation of CHK1 caused by gemcitabine is blocked by the addition of an ATR inhibitor (AZ20), but not Ataxia Telangiectasia Mutated (ATM) inhibitor (KU-55993). Next, we tested the effect of 10 nM gemcitabine in combination with AZ20, as well as KU-55993 and a DNA-PK inhibitor (NU7441), on cell viability. Consistent with the effect of inhibition of ATR on the activation of CHK1, the ATR inhibitor significantly reduced Ewing sarcoma cell growth in combination with gemcitabine (Figure 4H). In contrast, the inhibitors of ATM and DNA-PK did not impair cell growth when combined with gemcitabine.

**Figure 5:**
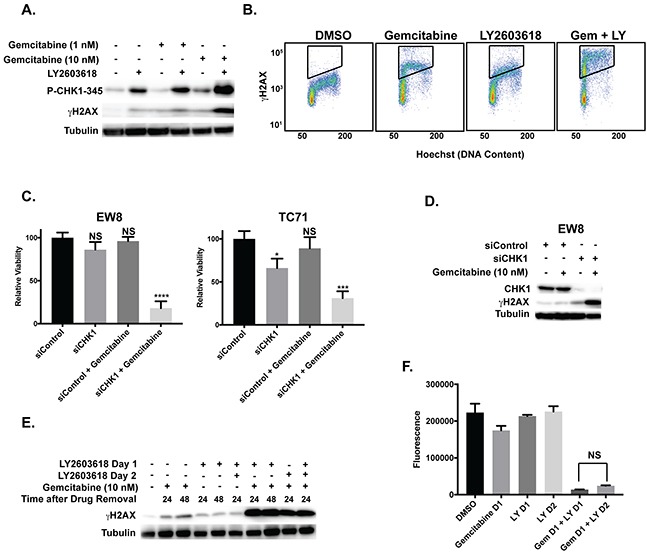
Induction of γH2AX by gemcitabine and LY2603618 **(A)** Western blot showing that treatment of EW8 cells with LY2603618 (250 nM) in combination with 10 nM gemcitabine, but not 1 nM gemcitabine, results in phosphorylation of H2AX. **(B)** The combination of LY2603618 (250 nM) with gemcitabine (10 nM) also increases the phosphorylation of H2AX relative to treatment with either drug as a single agent, as assessed using flow cytometry. **(C)** Relative viability of EW8 and TC71 cells treated with control siRNA, CHK1 siRNA, and the combination of each siRNA with gemcitabine (10 nM, 6 hours). Gemcitabine was added 24-hours after siRNA transfection. Cell viability was then assessed 18-hours after drug removal using the AlamarBlue Fluorescence Assay. ^*^ P-value< 0.05, ^***^ P-value< 0.001, ^****^ P-value< 0.0001 (1-way ANOVA, Dunnett's *post hoc* test). **(D)** Western blotting showing that the treatment of EW8 cells with the combination of gemcitabine and CHK1 siRNA, as described in **(C)**, results in phosphorylation of H2AX. **(E)** Western blot showing that treatment of EW8 cells with gemcitabine (10 nM) in combination with LY2603618, using a concurrent or staggered drug administration schedule, results in similar phosphorylation of H2AX. Cell lysates were collected at different time points after drug removal to ensure equivalent post-drug recovery periods. **(F)** EW8 cells were treated with gemcitabine (10 nM) for 6 hours on day 1 and LY2603618 (250 nM; 6 hours) on day 1 or day 2. Cell viability was then assessed 24 hours after drug removal using the AlamarBlue Fluorescence Assay. NS, not significant.

The dose-response experiments identified that a 6-hour treatment of 10 nM gemcitabine in combination with 250 nM LY2603618 is sufficient to kill Ewing sarcoma cells. Consistent with this cell viability data, the combination of LY2603618 (250 nM) with 10 nM gemcitabine, but not 1 nM gemcitabine, caused robust phosphorylation of H2AX, a marker of DNA damage and double strand breaks (Figure 5A) [52, 53]. We also evaluated the phosphorylation of H2AX using flow cytometry. Figure 5B shows that the combination of LY2603618 with gemcitabine increases the phosphorylation of H2AX relative to treatment with either drug as a single agent. Next, to use an orthogonal approach to block CHK1 function, we used siRNA to knockdown CHK1 and then treated cells with 10 nM gemcitabine for 6 hours. The combination of CHK1 siRNA and gemcitabine significantly reduced the growth of Ewing sarcoma cells (Figure 5C) and caused phosphorylation of H2AX (Figure 5D). Of note, although we did not observe significant toxicity with CHK1 siRNA alone at 48 hours after transfection, which is the time-point shown in Figure 5B, incubation for additional time resulted in a decrease in cell viability (data not shown).

Motano et al. demonstrated, using a variety of non-Ewing sarcoma cell lines, that addition of a CHK1 inhibitor (MK-8776) from 18-24 hours after a 6-hour incubation with gemcitabine induced significantly greater toxicity and γH2AX than if the two drugs were incubated concurrently for 6 hours [75]. However, we did not observe this effect in EW8 cells and the concurrent and staggered drug administration schedules resulted in similar phosphorylation of H2AX (Figure 5E) and cell viability (Figure 5F). Additionally, in other cell types, the combination of gemcitabine and a CHK1 inhibitor has been reported to cause aberrant entry into mitosis [76, 77]. In Ewing sarcoma cells, however, LY2603618 in combination with gemcitabine did not lead to abrogation of the cell cycle checkpoint or aberrant entry into mitosis (Supplementary Figure 8).

### Ewing sarcoma xenografts respond to the combination of gemcitabine and LY2603618

**Figure 6:**
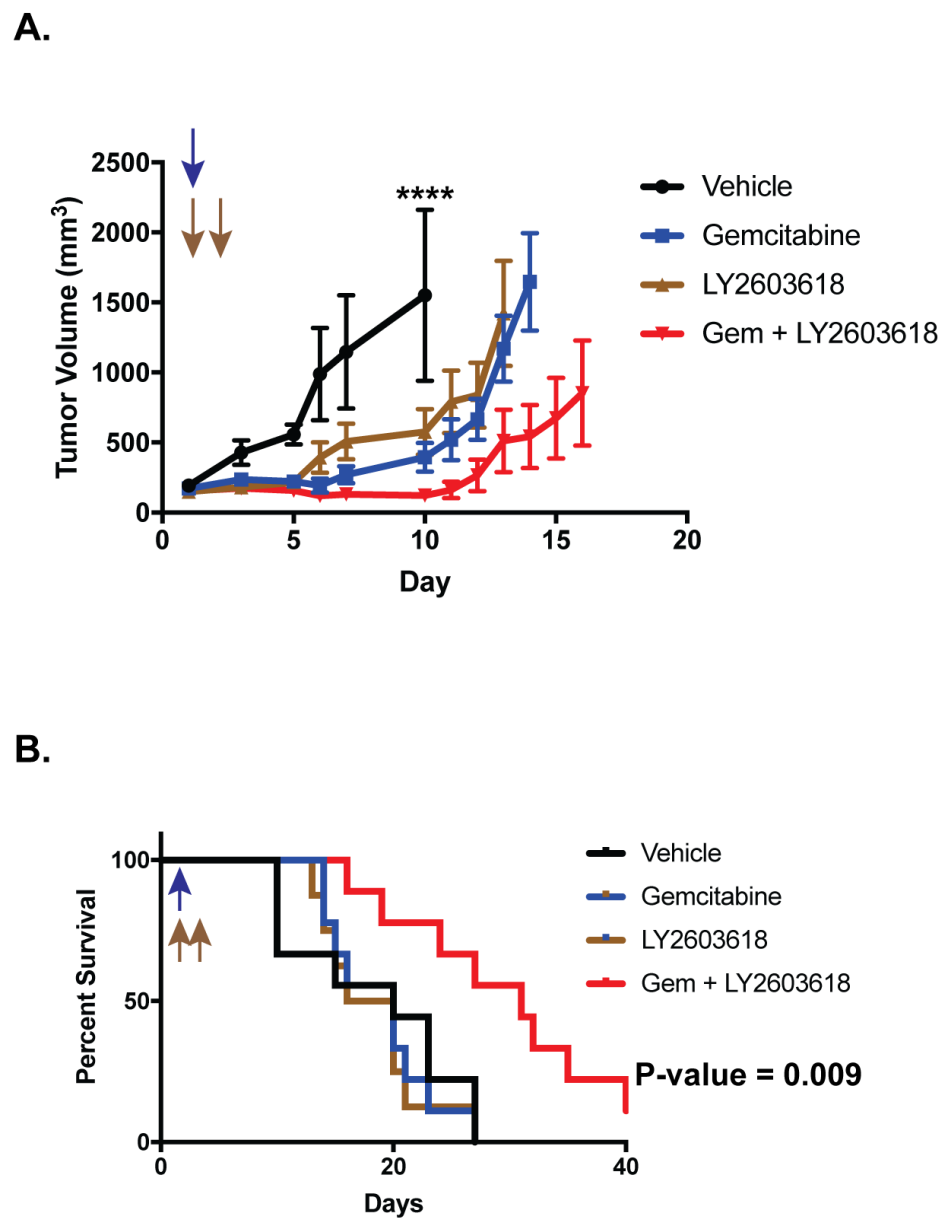
Ewing sarcoma xenografts respond to the combination of gemcitabine and LY2603618.TC71 cells were engrafted in nude mice After developing tumors, the mice were divided into four cohorts and treated with either vehicle, gemcitabine (150 mg/kg, intraperitoneal, once on day 1), LY2603618 (200 mg/kg, oral, once daily on days 1 and 2), and the combination of gemcitabine (day 1) and LY2603618 (day 1 and 2). **(A)** Tumor size was quantified using caliper measurements and tumor volumes were calculated using the equation volume = 0.5 × length × width^2^ (mean ± SD). Animals were sacrificed when a tumor reached 20 mm in any dimension. Growth curves for each drug treatment cohort are shown until mice were removed from that cohort due to tumor size. P-values were determined by 2-way ANOVA comparing the treatment curves to the vehicle curve through day ten, at which point the first mouse in the control group was sacrificed due to tumor size. Vehicle versus Gemcitabine + LY2603618, ^****^ P-value< 0.0001 **(B)** Kaplan-Meier survival curves for the different mouse cohorts. Log-rank (Mantel-Cox) test was used to calculate P-values comparing the survival curves.

We next tested whether gemcitabine and LY2603618 could inhibit the growth of tumor cells in a mouse xenograft experiment. NCr mice were subcutaneously injected with Ewing sarcoma (TC71) cells and allowed to develop measurable tumors. The mice were then divided into cohorts and treated with vehicle, gemcitabine (150 mg/kg, intraperitoneal, once on day 1), LY2603618 (200 mg/kg, oral, once daily on days 1 and 2) and the combination of gemcitabine (day 1) and LY2603618 (day 1 and 2). Figure 6A shows that there was a statistically significant difference in tumor volumes between the control and drug treated groups. Notably, there was also a significant difference in survival between the mice treated with the combination of gemcitabine and LY2603618 as compared to vehicle, or either drug alone (Figure 6B).

## DISCUSSION

Ewing sarcoma is treated with highly intensive, cytotoxic chemotherapy in combination with surgery and radiation [1, 78–80]. Despite aggressive therapy, the treatment outcomes are suboptimal, in particular for patients with metastatic disease. The overall survival of patients with metastatic and non-metastatic disease are ~20% and ~70%, respectively [80]. The current treatment of Ewing sarcoma is also associated with significant on- and off-treatment morbidities, including secondary malignancies, heart failure and renal toxicity [79]. Consequently, there is an unmet need in Ewing sarcoma to identify novel therapeutic approaches that will improve outcomes and reduce toxicity.

We previously used a stem cell model to identify that Ewing sarcoma cells are sensitive to inhibition of RNR [20, 21]. In this work, we demonstrate that Ewing sarcoma cells are also sensitive to aphidicolin, a drug that inhibits DNA polymerase and impairs DNA replication by an alternative mechanism than RNR [46, 47]. Clofarabine, which is a nucleoside analogue and inhibitor of both RNR and DNA polymerase, also reduced viability and induced apoptosis in Ewing sarcoma cells. However, clofarabine is a reversible inhibitor of RNR and we discovered that a short (6-hour) treatment with this drug, which more accurately reflects how this drug is administered in patients, did not significantly impact cell viability [41]. Gemcitabine, on the other hand, is an irreversible inhibitor of RNR and we identified that a short, 6-hour treatment with this drug is sufficient to induce apoptosis in Ewing sarcoma cells [42]. Moreover, inhibition of CHK1 function using a small-molecule inhibitor was synergistic with gemcitabine in Ewing sarcoma cells. Additionally, we also show that the combination of gemcitabine and a CHK1 inhibitor inhibits the *in vivo* growth of Ewing sarcoma cells in a xenograft model.

Treatment of Ewing sarcoma cells with gemcitabine results in the activation of CHK1 via ATR, which is a well-established upstream regulator of CHK1 [59, 81, 82]. As predicted, we also showed that Ewing sarcoma cells are sensitive to short (6-hour) drug treatments with gemcitabine in combination with an ATR inhibitor (AZ20). Although we did not observe that AZ20 caused toxicity when used as a single agent in a 6-hour drug treatment, we did identify, as reported by Nieto-Soler et al., that Ewing sarcoma cells are sensitive to AZ20 when used with longer incubations (data not shown) [25]. Interestingly, ATR inhibitors are known to reduce the levels of the RRM2 subunit of RNR via degradation of E2F1, a transcriptional activator of the *RRM2* gene [81]. Consequently, the toxicity of ATR inhibitors as single agents with Ewing sarcoma cells may, in part, be due to inhibition of RNR by reducing levels of the RRM2 subunit. We identified that the combination of gemcitabine with a CHK1 inhibitor (LY2603618) was more effective at inhibiting Ewing sarcoma cell growth than the combination of gemcitabine with an ATR inhibitor (AZ20). This difference in sensitivity between CHK1 inhibitor and ATR inhibitor in combination with gemcitabine may be explained by recent studies that have reported unexpected differences between the effects of ATR and CHK1 inhibitors. For example, Buisson et al. identified that CHK1 inhibitors induces cell death at a lower threshold of replication stress than ATR inhibitors [81]. In addition, the differential effects of the ATR inhibitor and CHK1 inhibitor when combined with gemcitabine could also be due to incomplete inhibition of CHK1 by ATR inhibitor. Wayne et al., for example, recently demonstrated that complete and sustained inhibition of CHK1 is necessary to activate a robust γH2AX induction and growth inhibition [83].

Other groups have also identified that Ewing sarcoma cells are uniquely sensitive to drugs that impair DNA replication or cause DNA damage in S-phase [25–31]. For example, Nieto-Soler et al. recently identified that Ewing sarcoma cells are sensitive to ATR inhibitors *in vitro* and *in vivo* [25]. This group also demonstrated that Ewing sarcoma cells exhibit high levels of endogenous DNA replication stress and elevated expression of the CHK1 protein [25]. However, the mechanism underlying the elevated levels of endogenous replication stress, as well as the sensitivity to drugs that increase replication stress or impair the response to DNA damage, is currently unclear [24]. Notably, unlike other driver oncogenes, EWS-FLI1 does not increase DNA replication, suggesting an alternative mechanism may be responsible [25]. EWS-FLI1 has been implicated as a regulator of multiple aspects of the cellular response to genotoxic stress. For example, SLFN11, a direct transcriptional target of EWS-FLI1, is overexpressed in Ewing sarcoma tumors and known to cause defects in checkpoint maintenance and homologous recombination repair [26, 84–87]. In addition, high levels of SLFN11 confer sensitivity of cancer cell lines to topoisomerase inhibitors, alkylating agents and DNA synthesis inhibitors, including gemcitabine [88]. An alternative, but not mutually exclusive, explanation is that haploinsufficiency of the *EWSR1* gene in Ewing sarcoma tumors could contribute to an impaired response to DNA damage [89, 90]. Or, EWSR1 translocations could mediate a dominant negative effect on endogenous EWSR1. Furthermore, recent germline sequencing of patients with Ewing sarcoma identified enrichment for mutations in genes involved with DNA damage repair [91]. From a mechanistic standpoint, the treatment of Ewing sarcoma cells with LY2603618 in combination with gemcitabine did not lead to abrogation of the cell cycle checkpoint or aberrant entry into mitosis, as has been reported in other cell types, and the mechanism of apoptosis induction is the focus of ongoing work.

Clinical trials testing gemcitabine, in combination with docetaxel, in patients with Ewing sarcoma have shown variable efficacy, which could be related to differences in gemcitabine doses between the regimens [92, 93]. Mora et al. used 1000 mg/m^2^ gemcitabine and showed an 80% (4/6) overall response rate (partial responses + complete responses) in patients with relapsed Ewing sarcoma [92]. However, a subsequent trial using a lower dose of 675 mg/m^2^ gemcitabine did not show similar efficacy as only 14% (2/14) of Ewing sarcoma patients showed a partial response [93]. Supporting a critical role for nucleoside dose in treatment response, a recent study of osteosarcoma patients treated with a higher dose of gemcitabine (900 mg/m^2^ versus 675 mg/m^2^) showed a significantly improved survival (1-year overall survival, 90.9 ± 8.7% vs. 38.5 ± 13.5%, P = 0.002) compared to patients who received the lower dose of gemcitabine [94]. A clinical trial testing single-agent cytarabine, which inhibits DNA replication but not RNR, in ten patients with relapsed or refractory Ewing sarcoma did not show efficacy [95, 96]. However, in this trial, cytarabine was administered as a single agent and at an intermediate dose level.

The combination of gemcitabine and a CHK1 inhibitor has been tested in several, early phase clinical trials [65, 66, 97–100]. For example, a recent Phase I trial demonstrated that LY2603618 is safe and well-tolerated when combined with gemcitabine (1000 mg/m^2^) [66]. Moreover, this drug combination was tolerable when administered as multiple, weekly cycles. Notably, in the mouse xenograft experiment (Figure 6), a single treatment with gemcitabine and LY2603618 resulted in a significant survival advantage compared to the control mice. Consequently, the efficacy of a more extended drug administration schedule, as well the efficacy of other CHK1 inhibitors combined with gemcitabine, will be a focus of future investigation [101].

In summary, we have identified that Ewing sarcoma cells are sensitive to gemcitabine, an irreversible inhibitor of RRM1. Moreover, combining gemcitabine with a CHK1 inhibitor is synergistic *in vitro* and significantly increases the efficacy of gemcitabine *in vivo* in a xenograft experiment. Overall, these results provide a rationale for the potential clinical translation of this drug combination for the treatment of Ewing sarcoma.

## METHODS AND MATERIALS

### Cell lines and culture

Cell lines were maintained at 37° C in a 5% CO_2_ atmosphere. The A673, TC32, TC71, SK-NEP, CADO and EW8 cell lines were kindly provided by Dr. Kimberly Stegmaier (Dana-Farber Cancer Institute, Boston, MA). The BJ-tert, HEK-293T, HT1080, RPE-tert, and U2OS cell lines were obtained from ATCC. Cells were grown in Dulbecco's Modified Eagle's Media (DMEM) supplemented with 10% FBS, 100 IU ml^−1^ penicillin and 100 μg ml^−1^ streptomycin. CHLA-9 cells were obtained from the Children's Oncology Group Cell Culture and Xenograft Repository (http://www.cogcell.org/) and cultured in Dulbecco's Modified Eagle's Media (DMEM) supplemented with 20% FBS, 100 IU ml^−1^ penicillin and 100 μg ml^−1^ streptomycin. Cell lines were authenticated by DNA fingerprinting using the short tandem repeat (STR) method.

### Chemical compounds

Chemical compounds were purchased from Sigma (gemcitabine, KU55933, and aphidicolin), Selleck Chemicals (LY2603618 and clofarabine), and Tocris (AZ20 and NU7441).

### Cell viability

Cell proliferation was measured using the resazurin (AlamarBlue) fluorescence assay [102]. Approximately 5 × 10^4^ cells were plated per well of a 96-well plate. Cells were treated with a range of drug concentrations for 6-72 hours. Fluorescence readings were obtained after adding the AlamarBlue reagent (Sigma) using a FLUOstar Omega microplate reader (BMG Labtech). IC50 values were then calculated using log-transformed and normalized data (GraphPad Prism 5.0). The combination index (CI) as a measure of drug synergy was determined using the method of Chou and Talalay with six drug concentrations at a fixed dose ratio [72]. The data were analyzed using the CompuSyn software (http://www.combosyn.com/).

### Clonogenic assay

Cells (500 cells/well) were plated in triplicate in a 6-well plate and allowed to adhere overnight. The cells were then treated with drugs, or vehicle, for six hours. The drugs were then removed and the cells were washed three times with PBS. The cells were allowed to grow for ~10-14 days and then fixed with 4% paraformaldehyde in PBS for 15 min. Colonies were stained for ten minutes with 0.5% methylene blue and 1% ethanol in PBS. After staining, the plates were washed four times with PBS. Colonies were then counted using an inverted Olympus CKX41 microscope.

### BrdU labeling

BrdU staining was performed according to the manufacturer's instructions provided with the Anti-BrdU (FITC-labeled, BD Biosciences, B44) antibody. Briefly, cells were incubated with 10 μM BrdU (Sigma-Aldrich) for 30 minutes at 37°C. The cells were then washed twice with 1%BSA/PBS and fixed with cold 70% ethanol. The DNA was then denatured using 2N HCl/Triton X-100. After neutralization of the acid, the cells were incubated with Anti-BrdU FITC antibody (BD Biosciences, B44, 20 μl of antibody per 1 × 10^6^ cells) for thirty minutes at room temperature. Flow cytometry was then performed using a BD Accuri C6 (BD Biosciences) instrument.

### γH2AX flow cytometry

Cells (3 × 10^5^ cells/well) were plated in a 6-well plate and allowed to adhere overnight. The cells were then treated with drugs, or vehicle, for six hours. Cells were then collected using trypsin, washed with PBS, and fixed for 15 minutes using 4% paraformaldehyde. Cells were then washed with PBS, re-suspended in cold 70% ethanol, and stored at −20°C overnight. Cells were then washed and re-suspended in 1% BSA/0.2% Triton X-100 in PBS, and incubated overnight at 4°C with the Alexa Fluor-647 anti-H2AX (pS139, BD Biosciences, 560447) antibody. Cells were then washed twice and re-suspended in PBS with 1 μg/ml Hoechst (ThermoFisher) dye. Flow cytometry was performed on a Becton Dickinson LSR II instrument.

### Xenograft

The Institutional Animal Care and Usage Committee at the University of Iowa approved the animal studies. The studies were conducted in adherence with the NIH Guide for the Care and Use of Laboratory Animals. Approximately 1.0 × 10^6^ TC71 cells were mixed with 30% matrigel and injected subcutaneously into the flanks of 6-week old, female NCr mice. After tumors were palpable (~100-200 mm^3^), mice were divided into cohorts (9 mice per cohort) and treated with drug or vehicle. In the clofarabine xenograft experiment, mice were treated with clofarabine (50 mg/kg) or vehicle by oral gavage daily for 5 days. Tumor volumes were measured periodically using calipers (volume = 0.5 × length × width^2^). All animals were sacrificed when the largest tumors in either the control or treatment groups reached 20 mm in any dimension. In the gemcitabine xenograft experiment, mice cohorts were treated with vehicle, gemcitabine (150 mg/kg, intraperitoneal, day 1), LY2603618 (200 mg/kg, oral gavage, days 1 and 2), or the combination of gemcitabine (150 mg/kg, intraperitoneal, day 1) and LY2603618 (200 mg/kg, oral gavage, days 1 and 2). LY2603618 was formulated for oral dosing in 16% Captisol (CyDex Inc) in 25 mM phosphate buffer, pH 4 [62, 70]. Tumor volumes were measured periodically using calipers (volume = 0.5 × length × width^2^). Animals were sacrificed when a tumor reached 20 mm in any dimension. GraphPad Prism was used to generate survival curves, which was determined by the time to 20 mm in any dimension.

### Genomics of drug sensitivity data analysis

The sensitivity of Ewing sarcoma cell lines to gemcitabine, compared to other cancer cell lines, was assessed using data from the Genomics of Drug Sensitivity in Cancer resource (http://www.cancerrxgene.org/) [57]. The IC50 values for cell lines treated with gemcitabine were log-transformed and a Mann-Whitney test was performed to compare the drug sensitivity of Ewing sarcoma cell lines versus other cell lines.

### Apoptosis assays

Caspase-3/7 activation was measured using the Caspase-Glo 3/7 Luminescence assay (Promega), according to the manufacturer's instructions. Annexin V was measured using a FITC Annexin V/Dead Cell Apoptosis Kit (ThermoFisher). The flow cytometry data were analyzed using FlowJo (v10.2).

### siRNA transfection

Cells (1.5-3 × 10^5^) were plated one day prior to transfection in six-well plates. Cells were transfected with siRNA using Lipofectamine RNAiMax (ThermoFisher Scientific) according the manufacturer's instructions. siCHK1 was a SMARTpool ON-TARGETplus reagent (GE Dharmacon). The sequence for siControl was 5’-UAGCGACUAAACACAUCAAUU-3’.

### Protein isolation and immunoblotting

Whole-cell extracts for immunoblotting were prepared by incubating cells in RIPA buffer (Boston BioProducts) plus protease and phosphatase inhibitors (Halt Protease & Phosphatase Inhibitor Cocktail, EDTA-free. ThermoFisher Scientific) for 20 min. Supernatants were collected following a 15 min centrifugation at 17,000 r.c.f. at 4°C. Protein concentrations were determined using the BCA reagent (Pierce). SDS-PAGE was used to separate proteins, which were then transferred to polyvinylidene difluoride membranes (Millipore). Antibodies to the following proteins were used in the immunoblots: phospho-Histone H2A.X (Ser139, Cell Signaling, #9718, 1:1000), phospho-Chk1 (Ser345, Cell Signaling, #2348, 1:1000), phospho-Chk1 (Ser317, Cell Signaling, #12302, 1:1000), phospho-Chk1 (Ser296, Cell Signaling, #12302, 1:1000), Chk1 (Cell Signaling, #2360, 1:1000), PARP (Cell Signaling, #9532, 1:1000), and tubulin (Proteintech, 66031-1, 1:2000).

### Cell cycle analysis

Cell cycle analysis was performed using the Click-iT EdU kit for flow cytometry (ThermoFisher Scientific). Cells were labeled with EdU for two hours and analysis was performed according to the manufacturer's instructions. Flow cytometry was performed on a Becton Dickinson LSR II instrument.

### Statistical analysis

Student's t-test was used to calculate P-values for the comparison of two groups. Analyses for more than two groups were conducted with a one-way analysis of variance (ANOVA) followed by Dunnett's multiple comparisons test to compare each group with the control group. P-values for the tumor volume measurements in the xenograft experiment were determined by 2-way ANOVA. The Log-rank (Mantel-Cox) test was used to calculate P-values comparing the survival curves in the mouse xenograft experiment. Statistical analyses were conducted using GraphPad Prism 5.0.

## SUPPLEMENTARY MATERIALS FIGURES AND TABLES


