# Supplementary material for: Inhibition of CHK1 sensitizes Ewing sarcoma cells to the ribonucleotide reductase inhibitor gemcitabine
Source: Oncotarget. 2017 Jun 28;8(50):87016–32. doi: 10.18632/oncotarget.18776 (PMC5675612; doi:10.18632/oncotarget.18776)
Supplement: Supplementary file 1 [file oncotarget-08-87016-s001.pdf]

Inhibition of CHK1 sensitizes Ewing sarcoma cells to the ribonucleotide reductase inhibitor gemcitabine

SUPPLEMENTARY MATERIALS

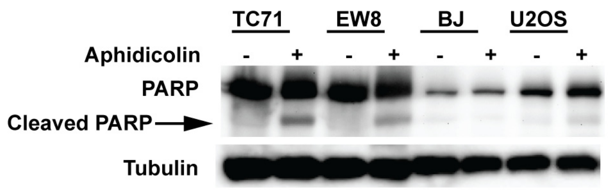

Supplementary Figure 1: Aphidicolin causes apoptosis in Ewing sarcoma cells. Western blot showing that treatment of Ewing sarcoma cells, but not U2OS osteosarcoma or BJ fibroblast cells, with aphidicolin (1 μM) results in cleavage of PARP-1.

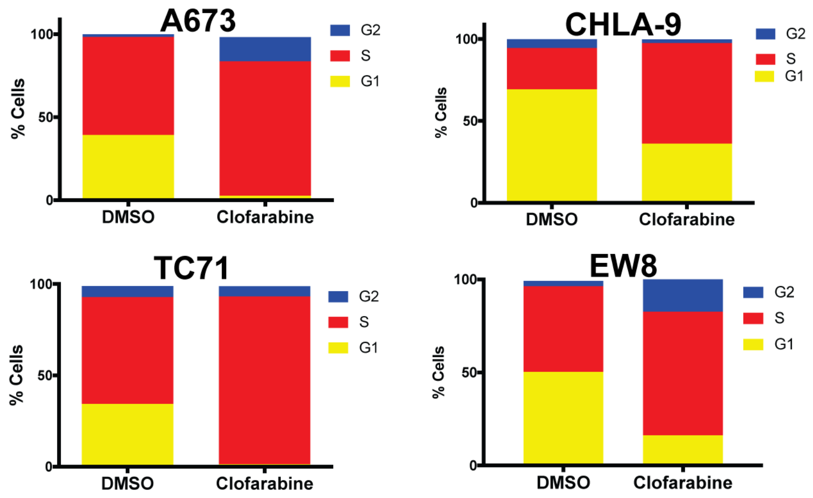

Supplementary Figure 2: Clofarabine impairs S-phase progression of Ewing sarcoma cells. Four Ewing sarcoma cell lines were treated with clofarabine (500 nM) for 24 hours and then fixed to analyze their cell cycle distribution using propidium iodide and EdU.

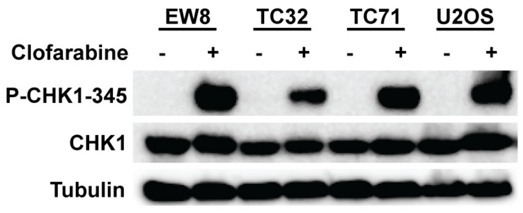

Supplementary Figure 3: Clofarabine causes phosphorylation of CHK1-345. Western blot showing that treatment of three Ewing sarcoma cell lines (EW8, TC32 and TC71) and an osteosarcoma cell line (U2OS) with clofarabine (500 nM) for 6 hours results in the phosphorylation of CHK1-345.

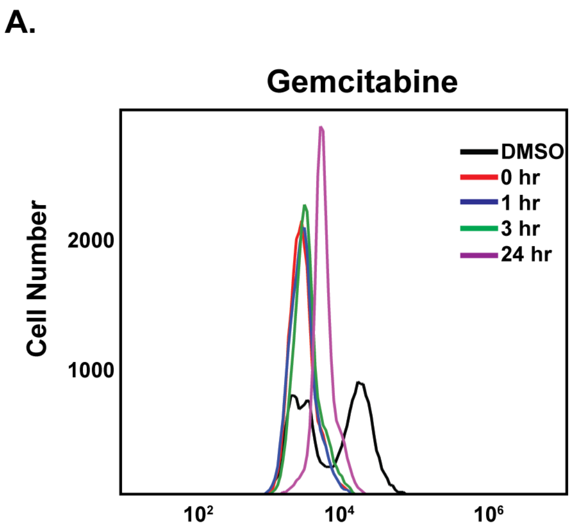

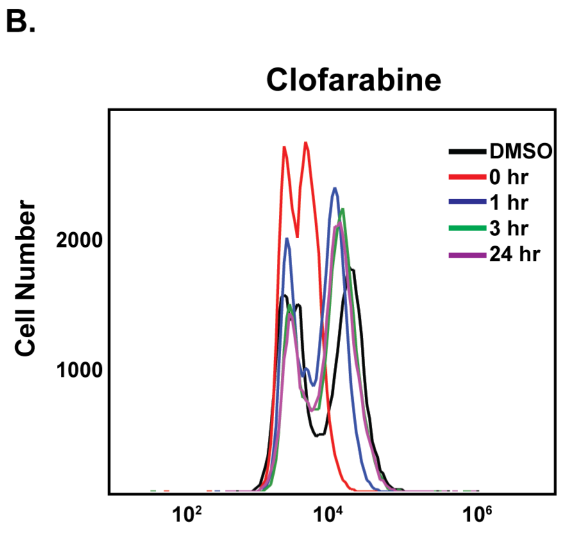

Supplementary Figure 4: Gemcitabine causes a sustained arrest of DNA replication in U2OS cells. **(A)** U2OS cells were treated with gemcitabine (500 nM) for 6 hours. BrdU incorporation was then quantified using flow cytometry at different time points after the removal of the drug. **(B)** As a comparison, U2OS cells were also treated with clofarabine (500 nM) for 6 hours. BrdU incorporation was then quantified using flow cytometry at different time points after the removal of the drug.

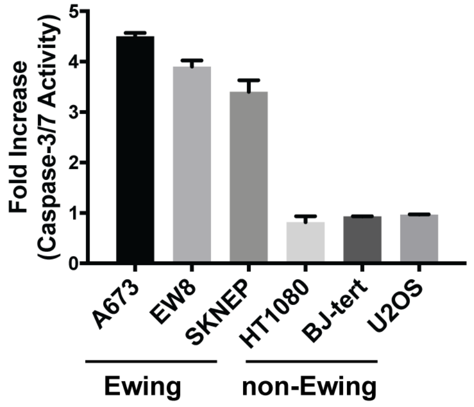

Supplementary Figure 5: A 6-hour gemcitabine treatment activates caspase 3/7 in Ewing sarcoma cells. Fold increase in caspase-3/7 activation in Ewing sarcoma and control cells lines treated with gemcitabine (100 nM) for 6 hours followed by a 42-hour recovery period. Fold change is relative to cells treated with DMSO. Figures are representative of three independent experiments. Data represent mean ± SD of three technical replicates.

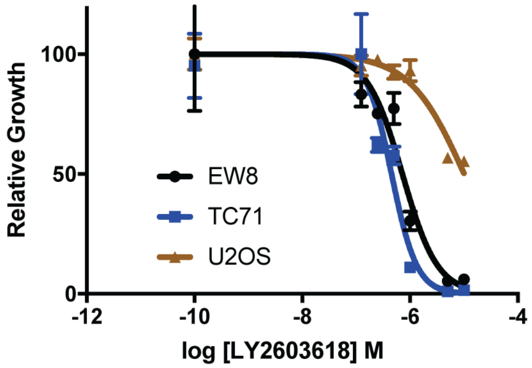

Supplementary Figure 6: LY2603618 inhibits the growth of Ewing sarcoma cells. Dose-response curves for two Ewing sarcoma cell lines and an osteosarcoma cell line (U2OS) treated with different concentrations of a CHK1 inhibitor (LY2603618) for 72 hours. Cell viability was assessed using the AlamarBlue Fluorescence Assay. The results are representative of two independent experiments. Error bars represent mean ± SD of three technical replicates.

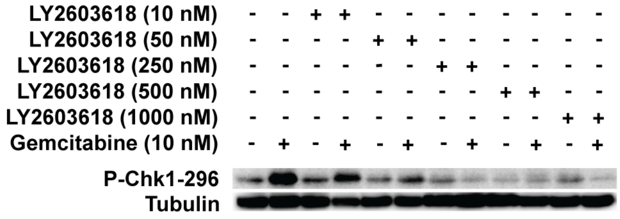

Supplementary Figure 7: LY2603618 causes dose-dependent inhibition of CHK1-296 auto-phosphorylation. Western blot showing that LY2603618 blocks the auto-phosphorylation of CHK1 (Ser296) that is induced by treating EW8 cells with gemcitabine (10 nM) for 6 hours.

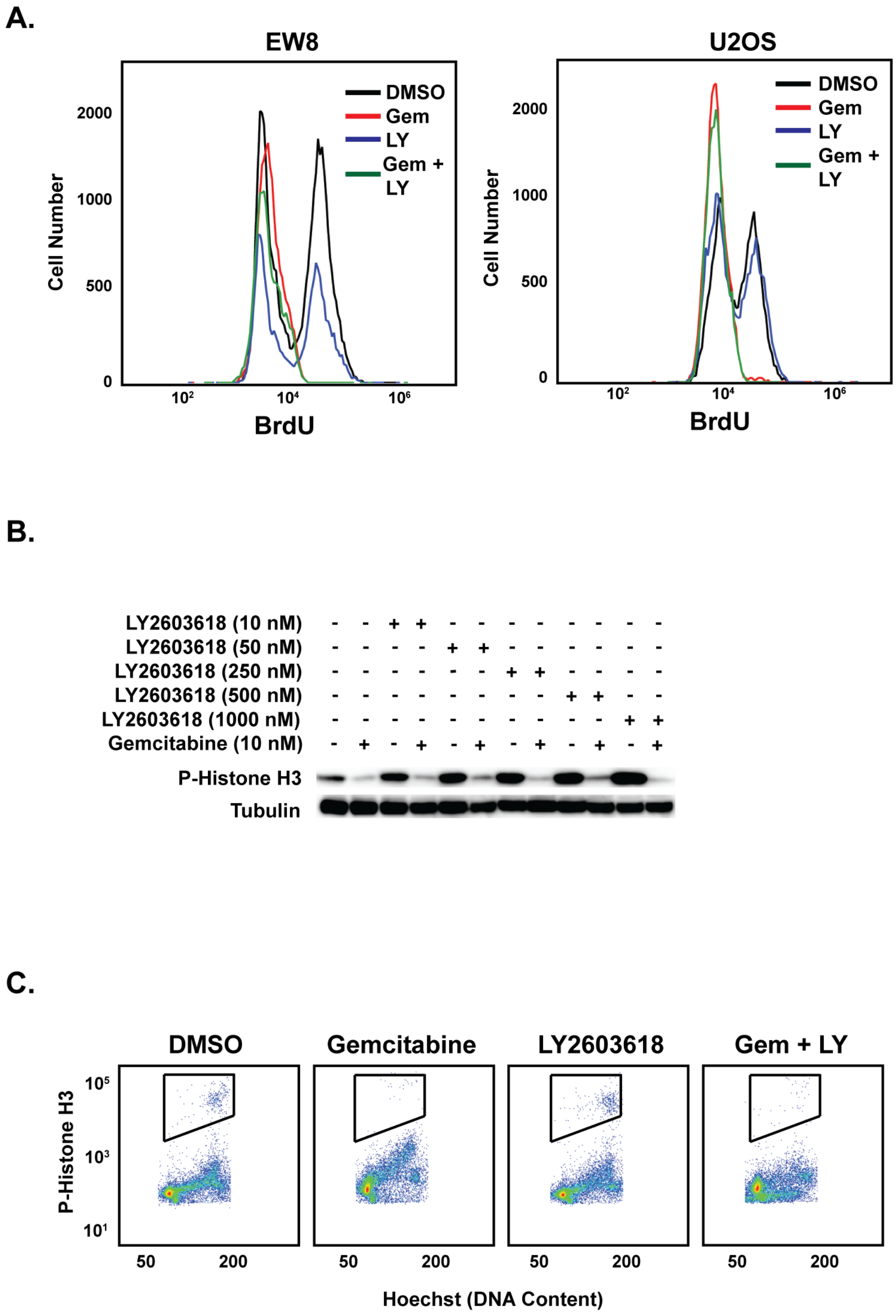

Supplementary Figure 8: The effect of LY2603618 on the DNA replication arrest caused by gemcitabine. (A) Quantification of BrdU incorporation into the DNA of EW8 and U2OS cells when treated with vehicle, gemcitabine (10 nM), LY2603618 (250 nM), and the combination of gemcitabine (10 nM) and LY2603618 (250 nM). **(B)** Western blot showing the effect of different concentrations of LY2603618 on the phosphorylation of Histone H3, in the presence and absence of gemcitabine (10 nM). **(C)** Quantification by flow cytometry of phospho-Histone H3 in EW8 cells treated with vehicle, gemcitabine (10 nM), LY2603618 (250 nM), and the combination of gemcitabine (10 nM) and LY2603618 (250 nM).
